# Breeding of a promising isogenic line of rice (*Oryza sativa* L.) variety ‘Koshihikari’ with low cadmium and brown spot (*Bipolaris oryzae*) resistance

**DOI:** 10.1270/jsbbs.24027

**Published:** 2024-11-15

**Authors:** Ritsuko Mizobuchi, Satomi Ohashi, Kengo Matsumoto, Yuya Ota, Tomohiro Yamakawa, Tadashi Abe, Satoru Ishikawa, Shinnosuke Ohmori, Yoshinobu Takeuchi, Akitoshi Goto, Kei Matsushita, Tomohito Ikegaya, Sayaka Kon, Nobuhiro Suzuki, Chikako Tsuiki, Utako Yamanouchi, Tsuyu Ando, Hiroyuki Sato

**Affiliations:** 1 Institute of Crop Science, National Agriculture and Food Research Organization, NARO, 2-1-2 Kannondai, Tsukuba, Ibaraki 305-8518, Japan; 2 Mie Prefecture Agricultural Research Institute, 530 Ureshinokawakita, Matsusaka, Mie 515-2316, Japan; 4 Institute for Agro-Environmental Sciences, National Agriculture and Food Research Organization, NARO, 3-1-3 Kannondai, Tsukuba, Ibaraki 305-8604, Japan

**Keywords:** *Oryza sativa* L., disease resistance, brown spot, *bsr1*, isogenic line, low cadmium, *osnramp5-2*

## Abstract

The *OsNRAMP5*-deficient rice (*Oryza sativa* L.) mutant (*osnramp5-2*), with a low grain cadmium (Cd) content, was registered as ‘Koshihikari Kan No. 1’ in Japan. Its agronomic traits are almost identical to those of ‘Koshihikari’ except for its low Cd content and its susceptibility to brown spot (BS) caused by *Bipolaris oryzae*. To restore BS resistance, we introduced the BS resistance quantitative trait locus (QTL) *bsr1* into it. The resulting isogenic line (IL) had higher resistance to BS than Koshihikari Kan No. 1 while retaining low grain Cd, with no significant difference from Koshihikari in grain yield or quality. This IL, which we named ‘Kanto IL 31’, can be used for breeding varieties with low Cd content and BS resistance.

## Introduction

Ingested cadmium (Cd) is toxic to the kidneys. To decrease the risk to human health, the joint FAO/WHO Expert Committee on Food Additives defined a provisional tolerable Cd intake of 25 μg kg^–1^ body weight per month (WHO 2011). Because cereals, including rice (*Oryza sativa* L.), are the main source of Cd in food, the Codex Alimentarius Commission set the maximum allowable level in polished rice at 0.4 mg kg^–1^ (Codex 2006). The loss of function of *OsNRAMP5*, a major Cd/Mn (manganese) transporter (Sasaki *et al.* 2012), reduces the Cd concentration in rice grains by inhibiting root uptake (Ishikawa *et al.* 2012). An *OsNRAMP5*-deficient mutant, *osnramp5-2*, was selected and registered as ‘Koshihikari Kan No. 1’ in Japan (Abe *et al.* 2017); since then, several low-Cd varieties have been bred by introducing *osnramp5-2* from it (NARO 2024).

The agronomic traits of Koshihikari Kan No. 1 are almost identical to those of ‘Koshihikari’ except for low Cd and Mn levels (Abe *et al.* 2017). Under conditions of Mn deficiency, rice plants are more susceptible to brown spot (BS) disease, caused by *Bipolaris oryzae* (Ohata and Kubo 1974). Consistent with its low Mn content, Koshihikari Kan No. 1 was reported to be more susceptible than Koshihikari in Mn-deficient paddy fields (Honma *et al.* 2017).

BS infects various tissues of rice plants (Ohata 1989). The rate of yield reduction caused by BS is reported over 20% (Chakrabarti 2001, Kamal and Mia 2009, Ou 1985). In Japan, BS infected 143 333 ha, the third-largest area after rice blast (765 183 ha) and sheath blight (533 182 ha), in 2021 (JPPA 2022). BS could become more serious under global warming because its ideal temperature range is around 30°C (Mizobuchi *et al.* 2016). Although very few quantitative trait loci (QTLs) for resistance to BS have been reported (Mizobuchi *et al.* 2016), we detected a major BS resistance QTL, *qBSfR11*, renamed *bsr1*, on chromosome (Chr.) 11 in field resistance tests of recombinant lines derived from crosses between the BS-resistant local resource ‘Tadukan’ and the susceptible ‘Hinohikari’. On this basis, we bred ‘Mienoyume BSL’, the world’s first practical variety with BS resistance, by introducing *bsr1* into the genetic background of the susceptible ‘Mienoyume’ (Matsumoto *et al.* 2021).

The objective of this study was to develop a variety with both low Cd and BS resistance by combining *osnramp5-2* and *bsr1*. We selected plants homozygous at *bsr1* and *osnramp5-2* from a cross between a *bsr1* near-isogenic line (NIL) with the Koshihikari genetic background and Koshihikari Kan No. 1. One isogenic line (IL) had a significantly lower BS disease score than Koshihikari Kan No. 1 while retaining a low grain Cd concentration, with no significant difference from Koshihikari in agronomic traits. We named this IL ‘Kanto IL 31’.

## Materials and Methods

### Development of Kanto IL 31

**Fig. 1.**
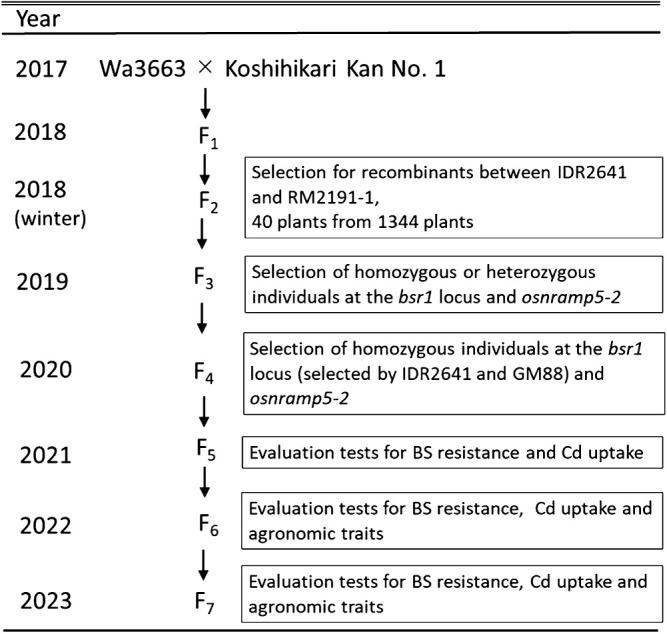
Scheme for development of Kanto IL 31.

The donor parents were line Wa3663, which has the BS resistance allele *bsr1*, and Koshihikari Kan No. 1 (Abe *et al.* 2017), which has *osnramp5-2* (Fig. 1). Wa3663 was selected as a BS-resistant line derived from a resistant line (R307-48-9) and has a chromosomal segment from Tadukan on Chr. 11 (1.4 Mbp defined by two DNA markers, IDR2641 and RM2191-1). R370-48-9 is a NIL (BC_3_F_4_) in which the major resistance QTL (*qBSfR11*, renamed *bsr1*) on Chr. 11, derived from the resistant *indica* cultivar Tadukan, had been introduced into the susceptible Koshihikari background (Matsumoto *et al.* 2021, Sato *et al.* 2015). The whole genome of R370-48-9 was surveyed by using 298 single-nucleotide polymorphism (SNP) markers distributed evenly across the 12 chromosomes (Nagasaki *et al.* 2010). We selected 40 F_2_ plants in which recombination had occurred within the *bsr1* region. Because *osnramp5-2* reduces BS resistance, we used lines without *osnramp5-2* for mapping of *bsr1* by testing BS field resistance.

Total DNA was extracted from fresh leaves as described (Hori *et al.* 2015) and used for PCR and electrophoresis as described (Mizobuchi *et al.* 2013), using the primer pairs listed in Supplemental Table 1.

### BS field resistance test

BS resistance was tested in the BS test paddy field of the Iga Agricultural Research Laboratory of the Mie Prefecture Agricultural Research Institute (Iga, Mie, Japan; 34°70ʹN, 136°13ʹE). Seedlings were transplanted in late May in 3 years (2021, 2022 and 2023) at 11 plants per row with 3 replications, at a spacing of 30 cm × 15 cm. Slow-release N fertilizer was applied at 7.5 g N m^–2^ at transplanting. As spreader plants, seedlings of a susceptible variety were inoculated with *B. oryzae* strain Iga-2 (Acc. No. 245177, MAFF Genebank) before transplanting around the rows but not within them. Because BS symptoms emerge gradually, resistance was assessed at maturity on a scale of 0 (no incidence) to 9 (severe) (Matsumoto *et al.* 2016).

### Uptake of Cd and Mn

Cd and Mn uptake was assessed in a greenhouse of the NARO Institute for Agro-Environmental Sciences (Tsukuba, Ibaraki; 36°03ʹN, 140°11ʹE) in 2021, 2022, and 2023. Plants of Kanto IL 31, Koshihikari Kan No. 1, and Koshihikari were grown in 1/5000-a Wagner pots filled with 3 kg of Cd-contaminated soil. Individual 1-month-old seedlings were transplanted into each pot in late May with 3 replications. Pots were flood-irrigated until the beginning of July, and then water was withheld to increase the bioavailable Cd concentration in the soil and to enhance Cd uptake by the plants (Ibaraki *et al.* 2009). The above-ground parts were harvested and divided into seeds and straw. Seeds were dehusked to brown rice (grains). Each sample was digested as described (Kuramata *et al.* 2022). The digests were filtered through a Millex-GP 0.22-μm PES filter (Merck Millipore) and analyzed for Cd and Mn by inductively coupled plasma–mass spectrometry (ICP–MS; ELAN DRC-e; Perkin Elmer Sciex) (Ishikawa *et al.* 2012).

### Yield trials, stress tests, and agronomic traits

Agronomic traits were evaluated in a paddy field of the NARO Institute of Crop Science (NICS; Tsukubamirai, Ibaraki; 36°01ʹN, 140°01ʹE) in 2022 and 2023. Kanto IL 31, Koshihikari Kan No. 1, and Koshihikari were sown on 14 April 2022 and 18 April 2023 and transplanted on 12 and 15 May, respectively. We raised 50 hills per plot with 2 replications at 3 seedlings per hill, at a spacing of 30 cm × 15 cm. Nitrogen fertilizer was applied at 8.0 g N m^–2^ at transplanting. Heading date, ripening date, culm length, panicle length, number of panicles per unit area, whole weight, yield of brown rice, lodging, grain quality, and eating quality were evaluated as described (Yamamoto *et al.* 1995). The sheath blight disease severity was assessed at ripening.

Blast resistance was evaluated at NICS (Tsukuba, Ibaraki; 36°03ʹN, 140°10ʹE), the Miyagi Prefectural Furukawa Agricultural Experiment Station (AES; Furukawa, Miyagi; 38°60ʹN, 140°91ʹE), and the Aichi Agricultural Research Center (Nagakute, Aichi; 35°21ʹN, 137°51ʹE). Sprouting resistance and high-temperature resistance were analyzed at NICS Tsukubamirai. Cold-temperature resistance was analyzed at the AES Furukawa and the Ishikawa Agriculture and Forestry Research Center (Kanazawa, Ishikawa, Japan; 36°64ʹN, 136°69ʹE). Blast resistance and tolerance to stresses (sprouting resistance, high temperature resistance, and cold temperature resistance) were evaluated as described (Yamamoto *et al.* 1995).

Statistical analysis was conducted in JMP v. 14.0.0. software (SAS Institute).

## Results

### Pyramiding of *bsr1* and *osnramp5-2* in Koshihikari genetic background

**Fig. 2.**
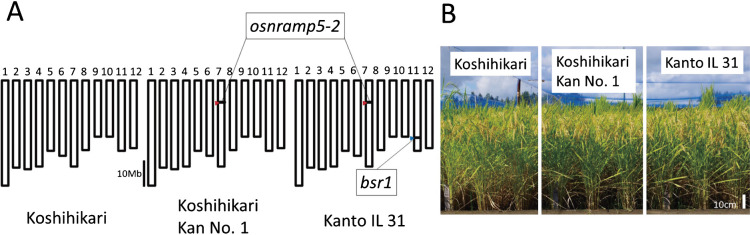
Genotypes and phenotypes of Koshihikari, Koshihikari Kan No. 1, and Kanto IL 31. A. Graphical genotypes (chromosomes 1–12). Arrowheads: red, position of *osnramp5-2*; blue, *bsr1*. B. Plants grown in a paddy field.

Among BS-resistant recombinants, a line selected by IDR2641 and GM88 (a 463-kb interval in the Nipponbare genome reference sequence) had the shortest chromosomal region from Tadukan. Therefore, we selected an IL with the same chromosomal region at the *bsr1* locus and with *osnramp5-2* and named it ‘Kanto IL 31’ (Figs. 1, 2).

### BS resistance of Kanto IL 31

**Fig. 3.**
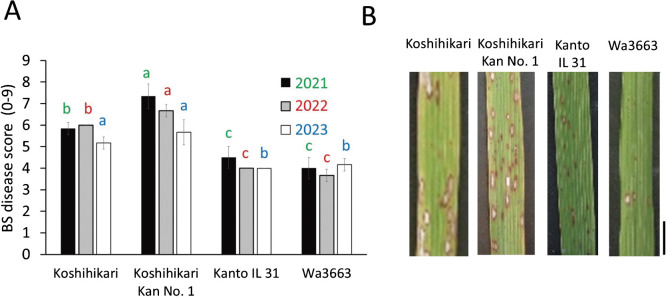
Evaluation of brown spot (BS) resistance of Kanto IL 31 by BS resistance field testing. A. Disease scores, ranked on a scale of 0 (no incidence) to 9 (severe) by eye at maturity. Data are means ± SD (11 plants × 3 repeats). In each year (2021; green letters, 2022; red, 2023; blue), the same letter indicates no significant difference (*P* < 0.05, Tukey’s HSD test). B. BS lesions on each plant type shown in A. Scale bar = 1 cm.

Koshihikari Kan No. 1 had a significantly higher BS score (5.7–7.3) than Koshihikari (5.2–6.0) (Fig. 3). Kanto IL 31 (4.0–4.5) and Wa3663 (3.7–4.2) had significantly lower BS scores than both. Thus, Kanto IL 31 retained BS resistance even in the presence of *osnramp5-2*.

### Cd and Mn concentrations in grains and straw of Kanto IL 31

The Cd concentrations in the grains of Kanto IL 31 (0.01–0.03 mg kg^–1^) and Koshihikari Kan No. 1 (0.01–0.04 mg kg^–1^) were extremely low, near the limit of detection (0.01 mg kg^–1^), but that in Koshihikari (0.38–1.04 mg kg^–1^) was much higher (Table 1). The Cd concentration in the straw was also much lower in Kanto IL 31 (0.02–0.14 mg kg^–1^) and Koshihikari Kan No. 1 (0.02–0.15 mg kg^–1^) than in Koshihikari (0.68–1.52 mg kg^–1^). These results suggest that Kanto IL 31 retains its low Cd content even in the presence of *bsr1*.

The Mn concentrations in the grains of Kanto IL 31 (3.36–5.17 mg kg^–1^) and Koshihikari Kan No. 1 (2.79–5.11 mg kg^–1^) were much lower than that of Koshihikari (17.47–28.45 mg kg^–1^; Table 1). Those in the straw of Kanto IL 31 (12.39–18.99 mg kg^–1^) and Koshihikari Kan No. 1 (13.47–17.53 mg kg^–1^) were also much lower than that of Koshihikari (173.43–186.49 mg kg^–1^; Table 1). These results suggest that Kanto IL 31 has a low Mn content because disruption of *OsNRAMP5* decreases Mn uptake, as in Koshihikari Kan No. 1.

### Agronomic traits of Kanto IL 31

There was no significant difference in the yields of brown rice among Koshihikari, Kanto IL 31, and Koshihikari Kan No. 1 (Table 2). There was no significant difference in 1000-grain weight or appearance. In 2022, because the degree of lodging was large owing to strong winds after heading, the scores of eating quality of Kanto IL 31 (–1.56), Koshihikari Kan No. 1 (–0.75), and Koshihikari (–1.13) were significantly lower than that of the Koshihikari reference crop (set to 0), which was grown in a different field for the eating test. In 2023, on the other hand, the scores of eating quality of Kanto IL 31 (–0.44), Koshihikari Kan No. 1 (–0.37), and Koshihikari (–0.19) were close to that of the Koshihikari reference crop. Therefore, it seems that there was no significant difference between Kanto IL 31, Koshihikari Kan No. 1, and Koshihikari in eating quality. The average culm length of Kanto IL 31 (90 cm) was significantly shorter than those of Koshihikari Kan No. 1 (94 cm) and Koshihikari (97 cm). There was no significant difference in heading date, ripening date, panicle length, number of panicles per unit area, whole weight, lodging degree, or sheath blight. There was no significant difference in blast resistance or tolerance to stresses (sprouting resistance, high temperature, cold temperature; Supplemental Tables 2, 3). In conclusion, there was no significant difference between Kanto IL 31 and Koshihikari in agronomic traits.

## Discussion

Kanto IL 31, with both *bsr1* and *osnramp5-2*, shows BS resistance even with low Cd and Mn contents due to *osnramp5-2*. Although rice is known to be susceptible to BS disease under Mn deficiency (Ohata and Kubo 1974), it is unclear why. Kanto IL 31 showed BS resistance even with low Mn. Therefore, resistance due to *bsr1* does not depend on increased Mn uptake. We assessed BS resistance in field tests and evaluated it at maturity. Initially, *bsr1* was identified as a QTL for BS resistance by the analysis of 18-day-old seedlings inoculated with *B. oryzae* (Sato *et al.* 2008). This evidence implies that *bsr1* functions from the seedling stage to maturity.

The culm length of Kanto IL 31 was 5 cm shorter than those of Koshihikari Kan No. 1 and Koshihikari. The difference was not so much, even though it was statistically significant. Kanto IL 31 has a chromosomal segment from Tadukan on Chr. 11 (463-kb defined by two DNA markers, IDR2641 and GM88), and 58 genes in this region of Nipponbare are predicted by the Rice Annotation Project Database (Ohyanagi *et al.* 2006). Because the genome of R370-48-9, which is a donor NIL (BC_3_F_4_) of *bsr1*, was checked by the whole genome SNP markers (Nagasaki *et al.* 2010), it is possible that the shortening of the culm length of Kanto IL31 depends on a pleiotropic effect of *bsr1* or other genes introduced from Tadukan by linkage. However, the genetic background of Kanto IL31 itself has not been confirmed. Therefore, it cannot be ruled out that there are unknown genetic factors in the background of Wa3663. Segregation analysis is needed in the progeny of Kanto IL31.

Mienoyume BSL, bred as a BS-resistant variety through the introduction of *bsr1*, was resistant to multiple isolates of BS fungus collected from several prefectures (Matsumoto *et al.* 2021). This result suggests that Kanto IL 31 will have BS resistance in multiple regions. Because Mienoyume BSL had a significantly lower BS disease score and a 28.8% higher yield in BS severe fields (Matsumoto *et al.* 2021), Kanto IL 31 will also be expected to have higher yield than Koshihikari Kan No. 1 under BS severe conditions. Cultivation testing of Kanto IL 31 for variety registration in Japan is now under way. In conclusion, our results suggest that *bsr1* is useful for breeding new varieties with both low Cd content and BS resistance.

## Author Contribution Statement

HS designed the research and bred the material until 2018. RM continued the breeding from 2019 to the present, and wrote the manuscript. HS and RM analyzed the data during the whole period. Thus, HS and RM are corresponding authors. SO, KM, YO, and TY performed BS resistance field testing and obtained phenotypic data for mapping of *bsr1*. TA and SI analyzed heavy metal concentrations of Kanto IL 31. SO, YT, AG, KM, TI, SK, and NS estimated agricultural traits of Kanto IL 31. CT, UY, and TA selected individuals with MAS.

## Supplementary Material

Supplemental Tables

## Figures and Tables

**Table 1.** Cd and Mn concentrations in grain and straw of Kanto IL 31, Koshihikari Kan No. 1, and Koshihikari

Tissue analyzed	Line or variety	Cd concentration (mg kg^–1^)				Mn concentration (mg kg^–1^)		
		Test year				Test year		
		2021	2022	2023		2021	2022	2023
Grain	Kanto IL 31	0.01 ± 0.00*	0.01 ± 0.00*	0.03 ± 0.01*		4.37 ± 0.56*	3.36 ± 0.13*	5.17 ± 0.65*
	Koshihikari Kan No. 1	0.01 ± 0.00*	0.01 ± 0.00*	0.04 ± 0.01*		4.83 ± 0.86*	2.79 ± 0.28*	5.11 ± 0.76*
	Koshihikari	1.04 ± 0.18	0.24 ± 0.06	0.38 ± 0.03		28.45 ± 2.71	17.47 ± 0.70	19.37 ± 2.02
Straw	Kanto IL 31	–	0.02 ± 0.00*	0.14 ± 0.03*		–	12.39 ± 2.76*	18.99 ± 1.19*
	Koshihikari Kan No. 1	–	0.02 ± 0.00*	0.15 ± 0.03*		–	13.47 ± 1.89*	17.53 ± 1.98*
	Koshihikari	–	0.68 ± 0.13	1.52 ± 0.20		–	173.43 ± 23.77	186.49 ± 47.37

Plants were grown in pots filled with soil from Cd-polluted paddy fields. Data are means ± SD (*n* = 3). Because the Cd concentration is affected by several conditions such as temperature and water management, the concentration in Koshihikari varied among test years. *Significant at 5% compared with Koshihikari (Student’s *t*-test).

**Table 2.** Agronomic traits of Kanto IL 31, Koshihikari Kan No. 1, and Koshihikari

Line or variety	Year	Heading date	Ripening date	Culm length (cm)	Panicle length (cm)	Number of panicles per unit area (No. m^–2^)	Whole weight (kg a^–1^)	Yield of brown rice*^a^* (kg a^–1^)	Yield percentage (%)	Lodging degree*^b^*	Sheath blight*^c^*	Grain quality		
												1000-grain weight*^a^* (g)	Appearance grain quality*^d^*	Eating quality*^e^*
Kanto IL 31	2022	7.26	8.31	88	18.3	516	149	50.3	115	4.5	2.0	21.5	6.3	–1.56**
	2023	7.28	9.02	91	19.9	448	161	53.0	98	2.0	0.5	22.0	6.0	–0.44
	Average	7.27	9.01	90*^f^	19.1	482	155	51.7	106	3.3	1.3	21.8	6.1	
Koshihikari Kan No. 1	2022	7.27	8.31	92	18.5	471	150	43.8	(100)	4.5	2.0	21.4	6.3	–0.75**
	2023	7.29	9.03	96	20.8	392	167	54.3	(100)	1.0	0.0	22.2	5.3	–0.37
	Average	7.28	9.01	94	19.7	432	158	49.1	(100)	2.8	1.0	21.8	5.8	
Koshihikari	2022	7.26	8.31	96	19.1	456	156	49.7	113	4.5	2.0	21.8	6.5	–1.13**
	2023	7.27	9.02	99	21.5	437	186	60.5	111	2.5	0.0	21.9	5.3	–0.19
	Average	7.26	9.01	97	20.3	446	171	55.1	112	3.5	1.0	21.9	5.9	

*^a^* Yield and 1000-grain weight were calculated from filled grains screened through a 1.8-mm-mesh sieve, at a moisture content of 15%. *^b^* Lodging degree was classified from 0 (standing) to 9 (lodged). *^c^* Disease severity was scored from 0 (no lesions) to 9 (totally dead). *^d^* Appearance grain quality was estimated in comparison with ordinary rice varieties and classified from 1 (excellent) to 9 (especially bad). *^e^* Eating quality shows the aggregate evaluation and was classified from +5 (excellent) to 0 to –5 (especially bad). The score of a reference crop of Koshihikari, which has good eating quality and was grown in a different field for this test, was set to 0. **Significant difference from Koshihikari reference crop at the 1% level (Student’s *t*-test). 2022 scores were lower than 2023 scores because of heavy lodging in 2022. *^f^* *Significant at 5% compared to Koshihikari (Student’s *t-*test).
