# Supplementary material for: Breeding of a promising isogenic line of rice (Oryza sativa L.) variety ‘Koshihikari’ with low cadmium and brown spot (Bipolaris oryzae) resistance
Source: Breed Sci. 2024 Nov 15;74(5):462–7. doi: 10.1270/jsbbs.24027 (PMC11780335; doi:10.1270/jsbbs.24027)
Supplement: Supplementary file 1 — Supplemental Tables [file 74_462_s1.pdf]

doi: 10.1270/jsbbs.24027

Supplemental Table 1. Primer pairs used for this study**.**

Name	Forward primer (5′–3′)	Reverse primer (5'–3')	Purpose
RM2191-1	GATAAGCATTTTAGAACACA	ACTAGACCAAGGAATTATTG	Genotyping for *bsr1* (SSR marker)
IDR2641	CGCATCTCAACCGTCTTCTG	TTATAGCACGCCGAGGTCAT	Genotyping for *bsr1* (indel marker)
GM88	GTCACGTGGCATCAGTTACG	CGTGCACATCAAGAACGCTA	Genotyping for *bsr1* (indel marker)
kmt2-F-w	TTTTTTTTTTTTTTTTTTTTGGTGTCGAGGCTGAGGCTGG		Genotyping for *osnramp5-2*
kmt2-F-m	GGTGTCGAGGCTGAGGTCGC		Genotyping for *osnramp5-2*
kmt2-R		GGCGCTGCTGATAAACATCGCC	Genotyping for *osnramp5-2*

Supplemental Table 2. Blast resistance of Kanto IL31, Koshihikari Kan No. 1, and Koshihikari.

Line or variety	Estimated genotype of blast resistance[a]	Field resistance to leaf blast[b]						Field resistance to panicle blast (Aichi Agricultural Research Center, 2023)[c]					
		NARO (2022)		NARO (2023)		Miyagi Pref. Furukara Agric. Expt. Stn. (2023)		Plot A		Plot B			
		Disease score	Evaluation	Disease score	Evaluation	Disease score	Evaluation	Heading date	Disease score	Heading date	Disease score	Average score	Evaluation
Kanto IL 31	+	5.3	ms	3.2	m	4.8	s	8.08	6.8	8.09	5.0	5.9	m
Koshihikari Kan No. 1	(+)	5.3	ms	2.7	mr	4.5	s	8.09	6.0	8.10	3.7	4.9	m
Koshihikari	(+)	5.5	s	2.7	mr	5.0	s	–	–	–	–	–	–

[a] Kanto IL 31 showed susceptible phenotype to four races (007: Ina86-137; 033.1: TH68-126; 035.1: TH68-140; 037.1: 24-22-1-1) by inoculation test. +, susceptible phenotype.

[b] The disease severity of 40–50-day-old plants was scored from 0 (no lesions) to 9 (leaves totally dead) on the basis of the diseased area. mr, moderately resistant; m, moderate; ms, moderately susceptible; s, susceptible.

[c] The disease severity of plants 30 days after heading was scored from 0 (no lesions) to 9 (totally dead). m, moderate; –, not tested.

Supplemental Table 3. Tolerance of Kanto IL 31, Koshihikari Kan No. 1, and Koshihikari to several stresses.

Line or variety	Sprouting resistance[a]					High-temperature tolerance[b]									Cold-temperature tolerance[c]							
	NARO (2022)		NARO (2023)			NARO (2022)				NARO (2023)					Miyagi Prefectural Furukara Agricultural Experiment Station (2023)				Ishikawa Agriculture and Forestry Research Center (2023)			
	Score	Evaluation	Score	Evaluation	Final evaluation	Heading date	Ave. temp. over 20 days after heading (°C)	% of white immature grains	Evaluation	Heading date	Ave. temp. over 20 days after heading (°C)	% of white immature grains	Evaluation	Final evaluation	Floret sterility (%) (Plot A)	Floret sterility (%) (Plot B)	Average (%)	Tolerance degree defined newly	Floret sterility (%) (Plot A)	Floret sterility (%) (Plot B)	Average (%)	Evaluation
Kanto IL 31	3.5	Strong	4.0	Moderately strong	Strong	8.10	27.5	56.4	m	8.06	29.9	89.0	m	m	42.0	46.0	44.0	6–5	35.1	44.7	39.9	Strong
Koshihikari Kan No. 1	3.5	Strong	3.5	Strong	Strong	8.11	27.5	49.3	m	8.08	29.9	87.1	m	m	26.0	40.0	33.0	>7	38.6	36.8	37.7	Strong
Koshihikari	3.5	Strong	4.0	Moderately strong	Strong	8.09	27.7	50.7	m	8.06	29.9	89.0	m	m	39.0	49.0	44.0	7	40.1	33.6	36.9	Strong

[a] Panicles harvested at maturity were dipped in hot water (37 °C) for 7 days, and sprouting resistance was judged on a scale from 2 (very strong) to 8 (very weak).

[b] Plants were grown in a vinyl greenhouse and white immature grains were measured with a Satake Rice Analyzer (RGQI 100A, Satake, Hiroshima, Japan). m, moderate.

[c] Plants were treated with cold water (19 °C). Tolerance was scored on a scale from 1 (very weak) to 9 (very strong).
